# Multiple Meningiomata in Cowden Syndrome

**DOI:** 10.7759/cureus.11469

**Published:** 2020-11-13

**Authors:** Vishal Aggarwal, AlHassan Mohmed, Ashwin Kumaria

**Affiliations:** 1 Neurosurgery, Queen's Medical Centre, Nottingham, GBR

**Keywords:** cowden syndrome, case report, meningiomata, srs, neurosurgery, gamma knife, pten

## Abstract

Cowden syndrome (CS) is a rare, congenital disease with associated cancers, but in a neurosurgical context is typically considered part of Lhermitte-Duclos. This misrepresentation is the likely cause of under-diagnosis. Furthermore, the presence of meningiomata has been described in CS patients but its absence as part of the condition’s major criteria suggest the correlation requires greater documentation. A 41-year-old woman with multiple cancers and a familial circumambience of CS was reviewed in clinic where multiple meningiomata were incidentally identified on MRI. Despite a lack of neurological impairment, and the general reluctance in treating the meningiomas of congenital disease with radiotherapy (RT), the patient underwent stereotactic radio-surgery (SRS) and at one-year post-procedure has reported no side effects or toxicity.

## Introduction

Cowden syndrome (CS) is a genetic disorder, and part of PTEN (phosphatase and tensin homolog) hamartoma-tumor syndrome (PHTS) [1]. PHTS are associated with disorganized growth of tissue-native-cells and whilst the progression of CS is highly variable, often characterized by multiple hamartomas of trans-dermal origin, it is the only PHTS disorder with a documented predisposition for systemic malignancies. PTEN encodes a dual phosphatase protein that negatively regulates the PI3K-Akt-mTOR pathway [2].

Whilst CS is estimated to affect 1 in 200,000 individuals, featuring a strong female predominance [3], the current neurosurgical involvement in CS is limited by its recognition in the context of Lhermitte-Duclos disease. Greater awareness amongst the neurosurgical community of CS as an independent pathology is required and may explain why even though the presence of meningiomata in CS has been eluded to, there is a lack of documented cases outlining this correlation. We present the following case to further emphasize the link between CS and concurrent meningiomas, and our treatment approach. Finally, we appraise the shortcomings of the current diagnostic criteria.

## Case presentation

A 41-year-old female was reviewed in the Neurosurgical Clinic upon referral from ENT colleagues. The patient was undergoing radiological surveillance for follicular thyroid cancer for which she had undergone a thyroidectomy 13 years ago. She underwent a limited scan which included her anterior skull base, and subsequently had a complete MRI scan with contrast, as described below. Of note in the patient’s medical history, she had the pathological involvement of her uterus, thyroid, and breast, all of which form parts of the major criteria for CS diagnosis [1,6] Interestingly, not only is the patient a mother to two daughters, both of whom have been genetically confirmed as CS-positive, but she was herself the daughter of a CS patient. Moreover, this awareness from our Genetic Services Division led to the initial investigations whereby the patient’s system-wide pathologies were brought to light.

The purpose of the patient’s present follow-up was to monitor the remission status of thyroid cancer; however, she was found to have three incidental meningiomata. The patient denied any headaches, seizures, or other problems that could be attributable to the meningiomata. She did not have any visual problems and was neurologically intact.

**Figure 1:**
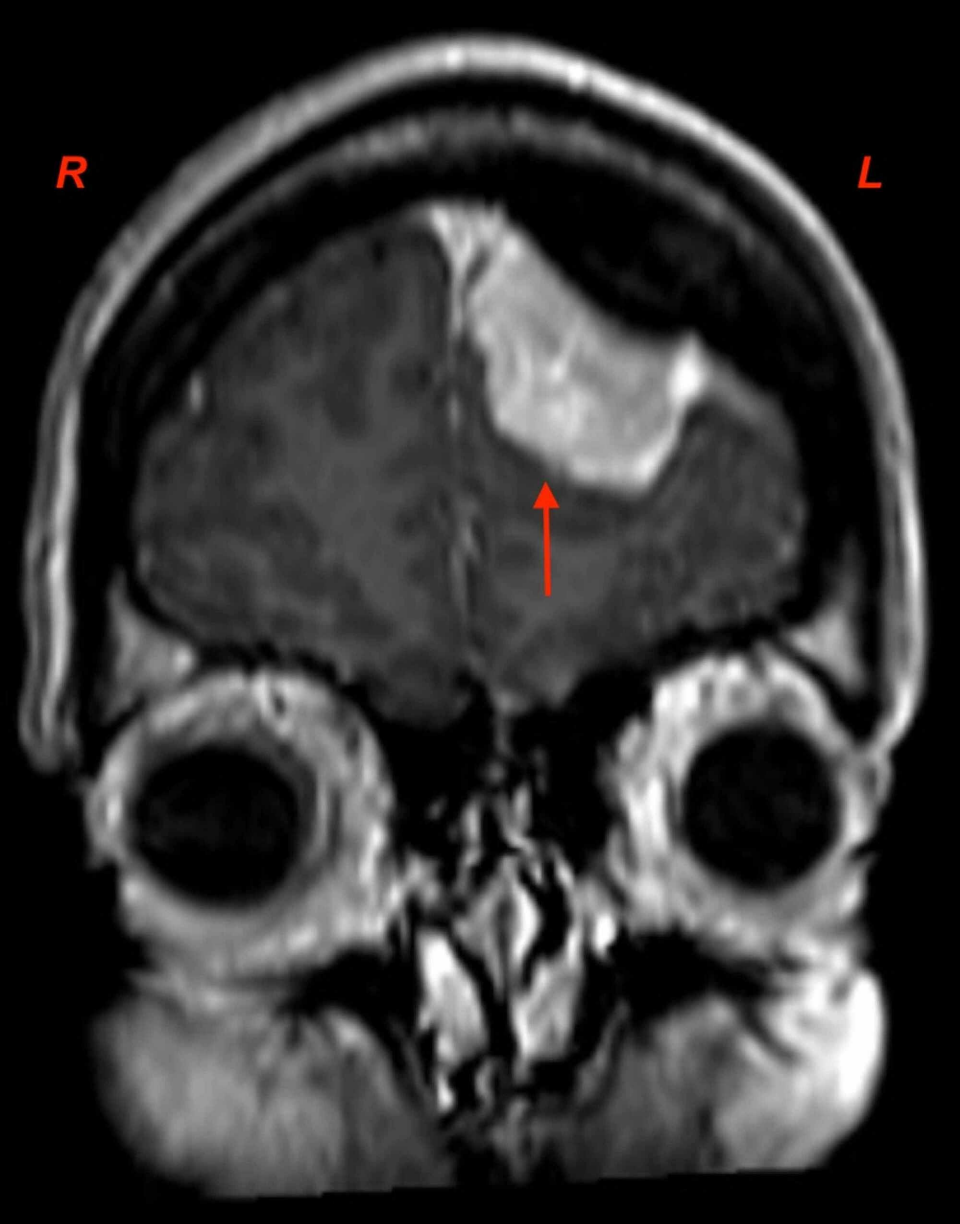
Coronal T1-weighted MRI-contrast scan demonstrating the left-sided, frontal meningioma. Parafalcine expansion is visible at depth.

**Figure 2:**
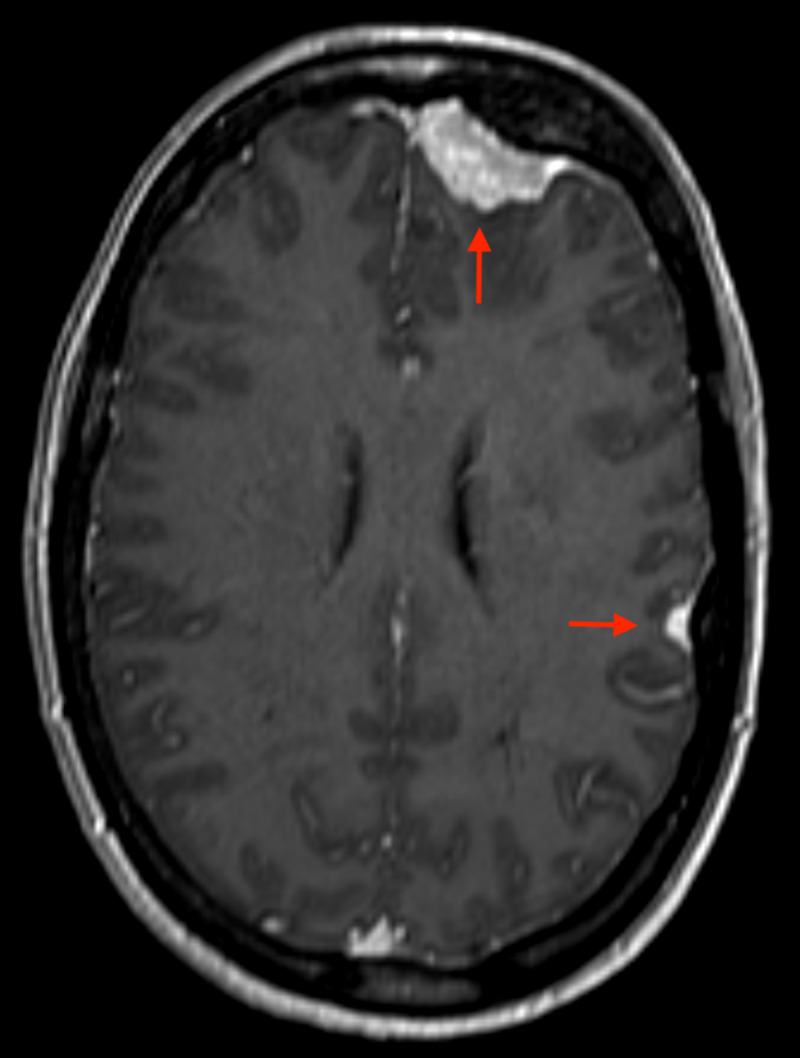
Axial T1-weighted MRI-contrast scan showing left frontal, and temporal meningiomata.

**Figure 3:**
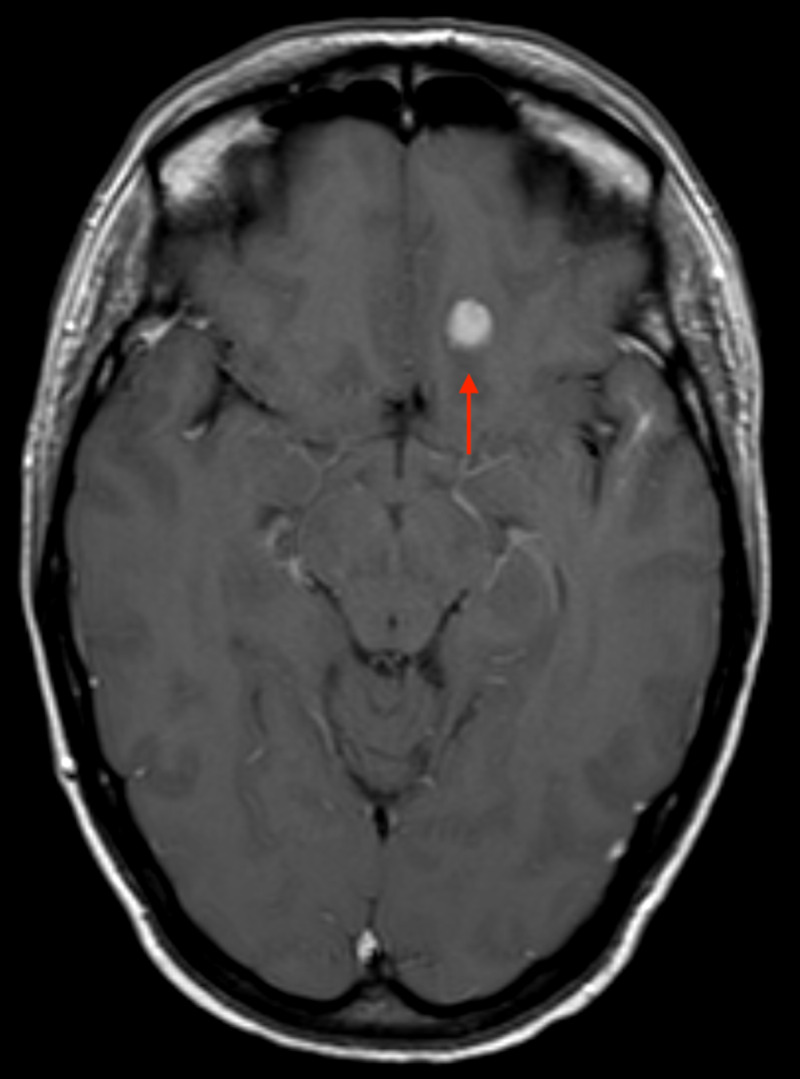
Axial T1-weighted MRI-contrast scan highlighting a solitary meningioma adjacent to the left-wing of the sphenoid.

An MRI brain scan in October 2018 revealed a small left frontal contrast-enhancing dural based lesion consistent with a meningioma of just over 3 cm x 1.5 cm, a left temporal convexity tiny meningioma, and a small left-sided sphenoid wing meningioma (Figures 1, 2, 3). The left frontal convexity meningioma was associated with a small amount of edema but there were no other concerning features. Her case was discussed at the NeuroOncology multidisciplinary team meeting, as is our practice for all newly diagnosed tumors. Owing to the multiplicity of lesions, the small individual volume size, and the small collective volume, she was considered a candidate for stereotactic radiosurgery. She remains well at one-year post Gamma Knife radiosurgery.

 

## Discussion

We present the case of a CS patient with meningiomata. Whilst meningiomas are the most common brain tumors, multiple meningiomas are found in only 1%-10% of patients [4], and are typically seen in certain congenital diseases, as well as acquired cases related to radiation and hormonal changes.

Of the familial syndromes, the most well-described is Neurofibromatosis Type 2 (NF2), an autosomal dominant tumor suppressor syndrome caused by a mutation in the NF2 gene that yields a meningioma rate of approximately 50% [5]. CS is also a familial, autosomal dominant disease but is typically multi-systemic and results in multiple hamartomas, often with associated mucocutaneous lesions, goiters, breast cancer, and gastrointestinal polyps [1,6,7]. It has incomplete penetrance and variable expressivity with a germline mutation in the PTEN tumor suppressor gene on chromosome 10q23.3. PTEN germline mutations are related to a wide range of brain tumors and whilst dysplastic gangliocytoma of the cerebellum, also known as Lhermitte-Duclos disease, is witnessed in 9% of CS cases and forms part of Pilarski et al.’s major criteria for CS diagnosis, meningiomas seen in 8% of CS cases do not [6,8]. Given the rarity of CS, with an estimated prevalence of 1:200,000 patients, the utility of an identified meningioma is questionable. Tumors with high incidence in the general population are arguably too non-specific for use in a diagnostic criteria, although the irony is that CS is most likely underestimated due to under-diagnosis in the first place.

Management of a solitary meningioma is often dictated by tumor size, location, symptoms, and patient preference, with surgical intervention often providing the means of resolution. However, patients with meningiomata are unable to achieve this without multiple open procedures, with considerable associated risks and potential morbidity [9]. The use of external beam radiation therapy in NF2 associated tumors remains controversial. Radiotherapy has been used in a subset of NF2 tumors that progress despite surgical treatment, or in individuals for whom there is an operative risk, but there is general hesitance in recommending radiation for NF2 patients with tumors of any size. Radiation should be used with caution in a setting of NF2 since secondary malignancies after RT treatment have been reported [10], although the correlation is not as strong as those seen in germline PTEN mutations [11]. It is therefore unsurprising that few publications exist describing the use of RT in patients with CS. Of the few documented cases, Tatebe et al. described the onset of skin desquamation, and near-total hair loss within the first week of whole-brain RT despite the modulation of intensity <10Gy [12]. Four months after therapy completion, the patient also developed emesis, ataxia, and headache with MRI showing both intracranial edema, and nodular enhancement; believed to be treatment-induced changes. In another report, a patient with CS was diagnosed with undifferentiated pleomorphic sarcoma of the breast, thought to be from adjuvant RT for breast cancer seven years prior [13]. Conversely Fernandez et al. document the case of a 35-year-old CS patient who underwent SRS at 15Gy which was not only well-tolerated, but at seven months post-RT devoid of side effects or toxicity [14].

Due to limited evidence, reports describing negative effects, and the reluctance to employ RT in NF2 patients, RT has been typically avoided in CS patients, thereby leaving clinicians with limited therapeutic options. When treating CS patients with RT, as is the case of those with meningiomata, precisely conformal, stereotactic techniques with minimal dose to normal structures should be utilized to decrease potential toxicity. Ultimately, the risk and benefits of RT should always be considered and a tailored approach should be employed with each patient. In our case, the decision to proceed with SRS was based on proactively managing the meningiomata. Despite their asymptomatic nature at the point of identification, the volume increase of meningioma is unpredictable and in waiting for burgeoning symptoms, the window of opportunity for performing SRS may have been lost.

## Conclusions

We report the successful use of intracranial SRS in the resection of a CS patient’s three meningiomata, with a positive outcome at one-year post-procedure. The elucidation of the patient’s family history, disease progression, and tumor’s growth predictability are paramount in formulating a diagnosis of CS, and assessing the best treatment strategy; where RT has typically been avoided in treating the meningiomata seen in congenital diseases, SRS may be a viable option. Finally, we suggest that meningiomas are included in the major criteria spectrum of CS.

## References

[1] Liaw D, Marsh DJ, Li J (1997). Germline mutations of the PTEN gene in Cowden disease, an inherited breast and thyroid cancer syndrome. Nat Genet.

[2] Marsh DJ, Coulon V, Lunetta KL (1998). Mutation spectrum and genotype-phenotype analyses in Cowden disease and Bannayan-Zonana syndrome, two hamartoma syndromes with germline PTEN mutation. Hum Mol Genet.

[3] Yakubov E, Ghoochani A, Buslei R, Buchfelder M, Eyüpoglu IY, Savaskan N (2016). Hidden association of Cowden syndrome, PTEN mutation and meningioma frequency. Oncoscience.

[4] Koech F, Orege J, Ndiangui F, Macharia B, Mbaruku N (2013). Multiple intracranial meningiomas: a review of the literature and a case report. Case Rep Surg.

[5] Tiwari R, Singh AK (2020). Neurofibromatosis Type 2. [Updated 2020 Aug 10]. https://www.ncbi.nlm.nih.gov/books/NBK470350/.

[6] Pilarski R, Burt R, Kohlman W, Pho L, Shannon KM, Swisher E (2013). Cowden syndrome and the PTEN hamartoma tumor syndrome: systematic review and revised diagnostic criteria. J Natl Cancer Inst.

[7] Nelen MR, van Staveren WC, Peeters EA (1997). Germline mutations in the PTEN/MMAC1 gene in patients with Cowden disease. Hum Mol Genet.

[8] Nieuwenhuis MH, Kets CM, Murphy-Ryan M (2014). Cancer risk and genotype-phenotype correlations in PTEN hamartoma tumor syndrome. Fam Cancer.

[9] Wong RH, Wong AK, Vick N, Farhat HI (2013;4). Natural history of multiple meningiomas. Surg Neurol Int.

[10] Blakeley JO, Evans DG, Adler J (2012). Consensus recommendations for current treatments and accelerating clinical trials for patients with neurofibromatosis type 2. Am J Med Genet A.

[11] Bergom C, West CM, Higginson DS (2019). The implications of genetic testing on radiation therapy decisions: a guide for radiation oncologists. Int J Radiat Oncol Biol Phys.

[12] Tatebe K, Chmura SJ, Connell PP (2017). Severe radiation toxicity associated with a germline PTEN mutation. Int J Radiat Oncol.

[13] Hatta N, Horita Y (2019). Undifferentiated pleomorphic sarcoma in a patient with Cowden syndrome after radiotherapy for breast cancer. J Dermatol.

[14] Fernandez C, Savard C, Farrell C, Shi W (2020). Successful stereotactic radiotherapy of meningiomas in a patient with Cowden syndrome: a case report. Chin Clin Oncol.

